# Do major life events influence physical activity among older adults: the Longitudinal Aging Study Amsterdam

**DOI:** 10.1186/1479-5868-9-147

**Published:** 2012-12-17

**Authors:** Margot A Koeneman, Mai JM Chinapaw, Marieke W Verheijden, Theo G van Tilburg, Marjolein Visser, Dorly JH Deeg, Marijke Hopman-Rock

**Affiliations:** 1 Body@Work, Research Center for Physical Activity, Work and Health, TNO-VU University Medical Center, Amsterdam, The Netherlands; 2 Department of Public and Occupational Health and EMGO Institute for Health and Care Research, VU University Medical Center, P.O. Box 7057, 1007, MB, Amsterdam, The Netherlands; 3 TNO The Netherlands organisation for applied scientific research, expertise center lifestyle, Leiden, The Netherlands; 4 Department of Sociology, VU University, Amsterdam, the Netherlands; 5 Department of Health Sciences, Faculty of Earth and Life Sciences and EMGO Institute for Health and Care Research, VU University, Amsterdam, the Netherlands; 6 Department of Epidemiology and Biostatistics and EMGO Institute for Health and Care Research, VU University Medical Center, Amsterdam, the Netherlands

## Abstract

**Background:**

Major life events are associated with a change in daily routine and could thus also affect habitual levels of physical activity. Major life events remain largely unexplored as determinants of older adults’ participation in physical activity and sports. This study focused on two major life events, widowhood and retirement, and asked whether these major life events were associated with moderate to vigorous physical activity (MVPA) and sports participation.

**Methods:**

Data from the first (1992–93) and second (1995–96) wave of the Longitudinal Aging Study Amsterdam (LASA), a prospective cohort study among Dutch adults aged 55 and older, were used. Change in marital status and employment status between baseline and follow-up was assessed by self-report. Time spent in MVPA (min/d) and sports participation (yes/no) was calculated based on the LASA Physical Activity Questionnaire. The association of retirement and widowhood with MVPA and sports participation was assessed in separate multivariate linear and logistic regression analyses, respectively.

**Results:**

Widowhood - N=136 versus 1324 stable married- was not associated with MVPA (B= 3.5 [95%CI:-57.9;64.9]) or sports participation (OR= 0.8 [95%CI:0.5;1.3]). Retired participants (N= 65) significantly increased their time spent in MVPA (B= 32.5 [95%CI:17.8;47.1]) compared to participants who continued to be employed (N= 121), but not their sports participation. Age was a significant effect modifier (B= 7.5 [90%CI:-1.1;13.8]), indicating a greater increase in MVPA in older retirees.

**Discussion:**

Our results suggest that the associations found varied by the two major life events under investigation. MVPA increased after retirement, but no association with widowhood was seen.

## Introduction

The importance of adopting and maintaining regular physical activity in old age is widely acknowledged [1,2]. Maintaining adequate levels of physical activity can prevent a decline in mobility and improve survival and wellbeing in older adults. Unfortunately, generally a decline in physical activity is observed after the age of 55 [2]. The limited amount of success in initiating and maintaining physical activity in older adults [3,4] shows a great need for knowledge of determinants of physical activity that can be targeted in interventions.

Major life events are associated with both positive and negative stress, which can affect health behaviour [5]. Major life events are associated with a change in daily routine [6] and, could therefore also affect habitual levels of physical activity [7,8]. Nevertheless, major life events remain largely unexplored as determinants of physical activity among older adults. Two major life events that frequently occur in late life and are known to cause distress are: change in marital status (widowhood) and change in employment status (retirement) [6,9]. Only one previous prospective study assessed the effects of interpersonal loss on physical activity adherence during a physical activity intervention for older adults [10]. Interpersonal loss was defined as death of a close family member, close friend or spouse, but no separate analyses were performed for widowhood. This intervention study observed that people experiencing interpersonal loss showed a decline in the number of home-based exercise sessions.

Some prospective observational studies assessed retirement and physical activity [11-16]. An increase in activity was reported after retirement [11,14]. Chung et al. [12] reported a decrease in physical activity for those who retired from a physically active job and an increase in physical activity for those who retired from a sedentary job. Slingerland et al. [15] concluded that retirement was associated with a decline in active transportation to and from work, while at the same time no increase in non-sport leisure time physical activity or sports participation was observed. Evenson et al. [13] and Touvier et al. [16] observed an increase in leisure-time physical activity in men and women who retired during follow-up, and observed at the same time an increase in time spent watching television. The cited studies show inconsistent results and only two of these studies [13,16] used validated physical activity questionnaires both at baseline and follow-up.

Therefore, our current study examined both widowhood and retirement as determinants of participation in moderate to vigorous physical activity (MVPA) and sports participation, assessed by a validated questionnaire at baseline and follow-up, in a prospective cohort study among Dutch older adults.

## Methods

### Sample

Data from the Longitudinal Aging Study Amsterdam (LASA), a prospective cohort study, was used [17]. The LASA study focuses on physical, cognitive and socio-emotional functioning during late life. Inclusion in LASA was based on a stratified sample of older adults born between 1908 and 1937 (initial age 55 and older). Stratified random sampling from municipal registries was used to comprise the original sample. The sample was weighted according to expected mortality at mid-term within each sex and age group, so that after five years equal numbers of men and women were expected to be alive in the ages 55–85 years. This design was chosen to leave sufficient participants to be examined after a period of 10 years. The sample was recruited in eleven towns in three regions. These three regions can be taken to represent differences in culture, religion, urbanization and aging in the Netherlands. Over 99% of the sample is of European descent. The original sample was recruited in 1992 by personalised introduction letter and an interviewer contacted them by telephone to schedule a face-to-face interview for the Living Arrangement and Social Networks of older adults (LSN). The LSN had a response rate of 65% (N=3.805). The 3.677 surviving participants of the LSN were re-approached eleven months later for a face-to-face interview for the first wave of the LASA study (1992–1993). Trained LASA-staff interviewed 3.107 men and women, aged 55 and older, at their home, yielding a response rate of 85%. More detailed information on data collection, inclusion and attrition is reported by Huisman et al. [18]. The LASA study was approved by the Ethical Review Board of the VU University Medical Center and informed consent was obtained from all respondents.

In our analyses we used the LASA interview data from the first (1992–93) and second (1995–96) wave. These waves had the highest number of participants and the highest absolute number of major life events compared to the subsequent waves. From now on the first wave will be referred to as ‘baseline’ and the second wave as ‘follow-up.’ At baseline trained LASA-staff interviewed 3.107 (response rate 85%) men and women, aged 55 and older, at their home. We have confined our sample to those participants living independently at baseline and for whom a full version of baseline and follow-up interview were available (N=2.480). The excluded participants were in institutionalised care (N= 119) or living in a monastery

(N=7). A further 501 participants did not provide data on both baseline and follow-up, most of the missing data was due to death prior to the follow-up or participants being too mentally or physically weak to be interviewed.

For the analyses on widowhood we included the participants of all birth years. For the analyses on retirement we included the participants who were between baseline and follow-up eligible for potential retirement, since retirement in the Netherlands is mandatory from the age of 65. These were the participants of the youngest years of birth ranging from 1928 to 1937. (See below and Tables 1 and 2 for a detailed description of the final sample). 

**Table 1:** Baseline characteristics stratified by widowhood

**Variable**	**Total sample (N=1.460)**	**Marriage continued (N=1.324)**	**Widowed (N=136)**	***p *value of between group difference**^**c**^
**Age**^a^	67.4 (7.9)	66.9 (7.8)	71.7 (7.8)	<.000
**Male gender**^b^	830 (57)	786 (59)	44 (32)	<.000
**Educational level**^b^				<.000
Low	510 (35)	440 (33)	70 (52)	
High	949 (65)	883 (67)	66 (49)	
Missing	1	1		
**Self-rated health**^b^				.180
Good	975 (67)	891 (67)	84 (62)	
Less than good	482 (33)	430 (33)	52 (38)	
Missing	3	3		
**No. of chronic diseases**^a^	1.2 (1.1)	1.2 (1.1)	1.5 (1.2)	.003
**MMSE** (0–30)^**a**^	27.4 (3.1)	27.5 (3.1)	27.1 (2.4)	.191
**CES-D** (0–60)^a^	6.3 (6.7)	6.1 (6.4)	8.6 (8.7)	<.000
Missing	10	10		

^a^ Values are expressed as mean (standard deviation).

^b^ Values are expressed as number (percentage of valid cases).

^c^ Independent *t*-test for age, cognitive functioning, depressive symptoms, number of self-reported chronic diseases, and χ^2^ test for gender, educational level and self-rated general physical health.

**Table 2:** Baseline characteristics stratified by retirement

**Variable**	**Total sample (N=186)**	**Employment continued (N=121)**	**Retired (N=65)**	***p *value of between group difference**^**c**^
**Age**^a^	58.7 (2.6)	59.0 (2.9)	58.1 (1.7)	0.015
**Male gender**^b^	124 (67)	74 (61)	50 (77)	0.030
**Educational level**^b^				.731
Low	31 (17)	21 (17)	10 (15)	
High	155 (84)	100 (82)	55 (85)	
**Self-rated health**^b^				.928
Good	151 (81)	98 (81)	53 (82)	
Less than good	35 (19)	23 (19)	12 (19)	
**No. of chronic diseases**^a^	0.7 (0.8)	0.8 (0.8)	0.6 (0.8)	.205
**MMSE** (0–30)^a^	28.4 (1.5)	28.3 (1.5)	4.6 (4.3)	.445
**CES-D** (0–60)^a^	4.8 (4.5)	4.6 (4.3)	5.1 (4.9)	452
Missing	1	1		

^a^ Values are expressed as mean (standard deviation).

^b^ Values are expressed as number (percentage of valid cases).

^c^ Independent *t*-test for age, cognitive functioning, depressive symptoms, number of self-reported chronic diseases, and χ^2^ test for gender, educational level and self-rated general physical health.

### Major life events

Information on *widowhood* was derived from a closed question assessing marital status with the categories 1) ‘never married’, 2) ‘married’, 3) ‘divorced’ and 4) ‘widowed’. Participants were classified as stable married (N=1.324) when they remained married from baseline to follow-up. Participants were classified as widowed when they lost their spouse by death between baseline and follow-up (N=136). Excluded were participants who married (N=3) or divorced (N=2) between baseline and follow-up, as well as those who were never married (N=109), divorced (N=121) or widowed at baseline (N=551). By comparing stable married participant to widowers only, we have excluded participants who indicated to live together with a partner without marriage (approximately 2% of the LASA sample). These participants are not included in the analyses.

Information on *retirement* was derived from two closed questions assessing employment status. Having a paid job at present was assessed with a yes/no question. Retirement was assessed with a closed question with the categories 1) ‘retired’, 2) ‘partial retirement’ and 3) ‘not retired.’ Participants were classified as stable employed (N=121) when they continued work from baseline to follow-up. Participants who had a paid job at baseline were classified as retired when did not have a paid job at follow-up and reported being in full retirement (N=65). Participants in partial retirement (N=11), who were not employed without indicating retirement (N=28) or who were involved in paid work (N=11) at follow-up were excluded from the sample. Participants retired or not employed at baseline were excluded as well (N=571). None of the participants classified as widowed experienced retirement and vice versa.

### Physical activity

*Physical activity* was assessed with the LASA Physical Activity Questionnaire (LAPAQ), a self-report physical activity questionnaire, based on the questionnaire by Voorrips et al. [19] and Caspersen et al. [20], designed especially for older adults. The LAPAQ recalls participation in daily physical activity and sport or exercise in the previous two weeks. The LAPAQ [21] showed good correlation with a 7-day diary (*r*=0.68, *p*<.001) and moderate correlation with a pedometer (*r*=0.56, *p*<.001). Respondents were asked how often and on average for how long in the previous two weeks they had engaged in each activity. First, we calculated activity in minutes per day by multiplying the frequency and duration of each separate activity in the previous two weeks and dividing the multiplied score by 14 (i.e., the total number of days). We then calculated time spent in moderate to vigorous intensity physical activity (MVPA) by summing all moderate to vigorous activities (walking, bicycling, heavy household activities and a maximum of two sports). The MVPA sum scores were expressed in minutes per day. Because of the highly skewed distribution of physical activity, including many scores of zero minutes on the selected activities, MVPA sum scores were converted to rank scores. Ranking recoded the sum scores into rank ordering from smallest to largest values. Equal sum scores remained ties and were assigned the mean score of those ties. Ranking of the MVPA scores was performed separately for the analyses of widowhood and retirement (Table 3 shows the distribution of rank scores stratified by widowhood and retirement). Therefore, we can only conclude on the relative change in rank of participants with respect to the other participants and not express change in minutes per day. This is in line with the purpose of the LAPAQ, namely to validly and reliably classify physical activity in older adults. Next, we assessed sports participation separately. Again because of a highly skewed distribution, due to many respondents who did not participate in sports, participants were classified as either participating in sports or not participating in sports. 

**Table 3:** MVPA and sports participation at baseline and follow-up stratified by widowhood and retirement

**Variable**	**Baseline**		**Follow-up**	
	**Marriage continued (N=1.324)**	**Widowed (N=136)**	**Marriage continued (N=1.324)**	**Widowed (N=136)**
**MVPA**^a^				
25th percentile	363	307	348	348
Median	722	581	727	585
75th percentile	1071	1004	1082	861
Missing	37	2	37	4
**Sports**^b^				
Yes (%)	723 (55%)	70 (52%)	486 (37%)	48 (35%)
No (%)	564 (43%)	64 (47%)	801 (61%)	84 (62%)
Missing	37	2	37	4
	**Baseline**		**Follow-up**	
**Variable**	**Employment continued (N=121)**	**Retired (N=65)**	**Employment continued (N=121)**	**Retired (N=65)**
**MVPA**^**a**^				
25th percentile	41	50	39	66
Median	94	86	77	117
75th percentile	137	143	128	154
Missing	1	1	1	1
**Sports**^**b**^				
Yes (%)	66 (55%)	40 (62%)	44 (36%)	29 (45%)
No (%)	54 (45%)	24 (37%)	76 (63%)	35 (54%)
Missing	1	1	1	1

^a^ Score is the ranked sum of walking, bicycling, heavy household activities, sports 1 and sports 2.

^b^ Score is the categorical variable yes/no sports participation.

### Covariates measured at baseline

*Self-rated general physical health* was measured on a 5-point scale ranging from ‘poor’ to ‘excellent.’ We have categorized these levels into ‘good’ (excellent and good) and ‘less than good’ (fair, sometimes good/bad and poor). *Self-reported number of chronic diseases* was assessed using a selection based on prevalence in the age group 55+ and functional consequences: 1. chronic non-specific lung disease (obstructive lung disease, asthma or chronic obstructive pulmonary disease); 2. cardiac disease; 3. peripheral arterial disease; 4. diabetes mellitus; 5. cerebrovascular accident or stroke; 6. osteoarthritis; 7. rheumatoid arthritis and 8. cancer [22]. In addition, respondents were asked whether they had any other chronic diseases (defined as a disease of which symptoms and/or treatment had been present for at least three months). A maximum of two other chronic diseases could be identified. *Depressive symptoms* were assessed using Radloff’s [23] self-report Center for Epidemiologic Studies Depression Scale (CES-D), a self-report depressive symptom rating scale validated for screening use in the Dutch population of older adults. The CES-D is a continuous measure (range 0 – 60) of increasing levels of depressive symptoms. The cut-off for clinically relevant depressive symptoms is set at a score of 16 or higher [24]. *Cognitive functioning* was assessed using the Mini-Mental State Examination (MMSE) [25]. The MMSE consists of 20 items and scores range from 0–30, higher scores indicating better cognitive functioning. A cut-off score for adequate cognitive function is set at a score of 26 [26]. Other covariates were *age* (in years), *gender* (male–female), and *educational level*. Educational level was assessed on a 9-point scale ranging from ‘elementary not completed’ to ‘university education’. We categorized this covariate into ‘low’ (elementary education or less), and ‘high’ (lower vocational education, general secondary education, intermediate vocational education, tertiary education or higher vocational education) levels of education.

### Statistical analyses

We compared baseline values of the widowed/retired group to the non-event group using the independent t-test for the variables age, cognitive functioning, depressive symptoms, number of self-reported chronic diseases and MVPA. A χ^2^ test was used for the variables gender, educational level, self -rated general physical health and sports participation.

Covariates were selected and tested for their potential confounding influence [27]. Demographics variables (age, gender, educational level), physical variables (self-rated general physical health and self-reported number of chronic diseases) and psycho-cognitive variables (depressive symptoms and cognitive functioning) were considered as confounder when: 1) they were significantly associated (*p* < 0.2) with the outcome measure (MVPA and/or sports participation at follow-up adjusted for baseline activity) as well as the major life event (widowhood/retirement) and when 2) the B-value (linear regression coefficient after ranking) of MVPA and/or the odds ratio of sports participation changed by more than ten percent when the potential confounder was added to the model (change-in-estimate using [100*(current model – previous model)/ absolute value of previous model]. In the case of multiple confounders, the combined effect of potential confounders was assessed using the same criterion, i.e., a variation of at least ten percent of the B after adjustment for the confounders entered into the model in blocks. In the first block demographic variables were entered, in the second block physical variables were entered and the third block consisted of psycho-cognitive variables [28].

Multivariate linear regression analyses was used to study the association between the selected major life event (widowhood or retirement) and follow-up MVPA, adjusted for baseline MVPA and confounders. Multivariate logistic regression analyses was used to study the association between the selected major life event (widowhood or retirement) and sports participation, adjusted for baseline sports participation and confounders. The significance level for the analyses of the main effect was set to an alpha of .05 or lower.

Effect modification by gender, age and educational level was examined by adding an interaction term between major life event (widowhood or retirement) and gender, age and educational level respectively, into the final model. The significance level for the interaction term was set to an alpha of .10 or lower [29]. Statistical analyses were conducted using SPSS 17.0.

## Results

Table 1 presents the baseline characteristics for the analyses of widowhood. From baseline to follow-up, 136 participants had a transition from being married to widowhood and 1.324 participants continued their marriage. Participants reporting widowhood were older, more frequently female and reported more chronic diseases and depressive symptoms than those with an unchanged marital status.

Table 2 presents the baseline characteristics for the analyses of retirement. From baseline to follow-up 65 participants retired. From baseline to follow-up 121 participants remained unchanged in employment status. Retired participants were younger and more often male than participants continuing their job.

Rank scores of MVPA and sports participation at baseline and follow-up are reported in Table 3. Widowed participants were less likely to spend time in MVPA (*p*= .04) at baseline compared to participants whose marriage continued; no difference was observed for participation in sports at baseline. With regard to retirement, time spent in MVPA at baseline and participation in sports did not significantly differ between those who retired and those who continued employment.

We identified four confounders of the relationship between widowhood and MVPA (i.e. age, subjective health, number of chronic diseases and CES-D score) and six confounders of the relationship between widowhood and participation in sports (i.e. age, gender, subjective health, number of chronic diseases, CES-D score and MMSE score). Gender was the only confounder of the relationship between retirement and MVPA and participation in sports.

Multivariate linear regression analyses showed that recent widowhood was not associated with MVPA (B= 2.70 [95%CI:-58.8;64.2]) or sports participation (OR= 0.9 [95%CI:0.6;1.4]), after adjustment for confounders (Table 4). We observed no significant effect modification by age, or gender. Educational level was a significant effect modifier for the association of widowhood with sports participation (OR= 2.2 [90%CI:1.0;4.1]). When analysing participants in the low and high education group separately, the association between widowhood and sports participation was not significant (high education group OR= 1.1 [95%CI:0.6;2.0], low education group OR= 0.6 [95%CI:0.3;1.2]). 

**Table 4:** Association between widowhood or retirement and MVPA and Sports

**Event**	**Outcome**	**Model 1**	**Model 2**	**Model 3**	**Model 4**
**Widowhood**	**MVPA**^**§**^	−66.9 (−129.9;-3.8)*	−11.9 (−73.6;49.9)	−5.1 (−66.4;56.2)	2.7 (−58.8;64.2)
Model 1	adjusted for baseline activity				
Model 2	Model 1 + age				
Model 3	Model 2 + subjective health and number of chronic diseases				
Model 4	Model 3 + CES-D				
**Widowhood**	**Sports**^¶^	1.0 (0.7;1.5)	0.9 (0.6;1.4)	0.9 (0.6;1.4)	0.9 (0.6;1.4)
Model 1	adjusted for baseline activity				
Model 2	Model 1 + age and gender				
Model 3	Model 2 + subjective health and number of chronic diseases				
Model 4	Model 3 + CES-D and MMSE				
**Retirement**	**MVPA**^**§**^	28.6 (13.9;43.2)**	32.5 (17.8;47.1)**	-	-
Model 1	adjusted for baseline activity				
Model 2	Model 1 + gender				
**Retirement**	**Sports**^**¶**^	0.7 (0.4;1.4)	0.6 (0.3;1.3)	-	-
Model 1	adjusted for baseline activity				
Model 2	Model 1 + gender				

**§**MVPA: B + (CI 95%).

**¶**Sports: OR+ (CI 95%).

* p<0.01.

** p< 0.001.

Retirement was significantly associated with MVPA (B= 32.5 [95%CI:17.8;47.1]) after adjustment for confounders (Table 4). The relative position in the ranking of MVPA scores was approximately 18% higher in the retired participants compared to those with continued employment. We observed no effect modification by gender or educational level. Age was a significant effect modifier (interaction age * retirement= 7.5 [90%CI:-1.1;13.8]), indicating a stronger association of retirement and MVPA with increasing age.

Multivariate logistic regression analyses showed that retirement was not significantly associated with sports participation (OR= 0.6 [95%CI:0.3;1.3]), after adjusting for confounders (Table 4). We observed no significant effect modification by age, gender or educational level.

## Discussion

The limited success in getting and keeping older adults physically active shows a great need for knowledge of determinants of physical activity. Even though a variety of possible determinants have been identified (e.g., characteristics of the individual, of the social and physical environment), major life events remained largely unstudied as possible motivators or barriers of physical activity in older adults. The current study examined the association of two major life events, namely ‘widowhood’ and ‘retirement’ with MVPA and sports participation among older adults. MVPA increased in those who retired compared to those who continued employment, but no influence of widowhood was observed. Furthermore, we observed no association between either major life event and sports participation.

The finding of no difference in MVPA or sports participation between those who lost their partner compared to those whose marriage continued was surprising, since we had hypothesized that a major life event, like widowhood could induce a change in daily routine and disrupt physical activity habits and result in a decline in physical activity [30-32]. A possible explanation is that widowhood may have acute and short-term effects on physical activity, but that these effects had subsided by the time of the follow-up assessment. Wilcox and King [10] showed a decline in adherence to an exercise intervention after interpersonal loss, suggesting immediate short-term effects on exercise behaviour. However, interpersonal loss may have a different impact on organised exercise sessions - as observed in the aforementioned intervention study - compared to daily physical activity, like biking or walking for transport [33]. Alternatively, in the period preceding widowhood, individuals may already adapt their physical activity to a lower level because of caring commitments. Assessing caring commitments and exact time of widowhood could provide additional insights in physical activity patterns. In this study, we were not able to examine such pre-widowhood adjustment. Future research is needed to explore the effects of widowhood on different aspects of physical activity, e.g. supervised versus unsupervised exercise and changes preceding a major life event.

As expected, retirement was significantly and positively associated with physical activity. Retired participants significantly increased their time spent in MVPA compared to employed participants. Contrary to our hypothesis no significant association with sports participation was observed. One previous study, similar to our design, used validated physical activity questionnaires both at baseline and follow-up and assessed sports participation separately [13]. This study showed an increase in sports participation, as assessed on a 5-point scale. A possible explanation for our contrasting finding may lie in our assessment of sports participation. To study the association between major life event and sports participation we have classified participants as either participating or not participating in sports. This classification allowed us to assess changes in the uptake or termination of sports participation but was not sensitive enough to detect an increase or decrease in time spent in established sports participation. Another explanation could be the cause of retirement. The retirees in our study population were on average 58 years of age, well below the 65 years mandatory retirement age. A recent study showed that early retirees (aged 55–58) were less likely to have a good perceived health, and health reasons may have been the reason for retirement [27]. This could explain our finding of the stronger association of retirement and MVPA with increasing age. Previous work from Lahti et al. [14] supports this view. Their study shows that contrary to older-age retirees, disability retirees did not increase their physical activity from baseline to follow-up. Future research is needed to explore the effects of various reasons for retirement on change in physical activity and sports participation. This may also include the physical burden of work and the economic status of the jobs, for these are factors that could possibly influence the need and opportunity for early retirement. Alternatively, like we proposed for widowhood, individuals who anticipate their retirement may have started to adjust their level of MVPA, so that we could not observe change in our study.

Since we have no data available on work-related physical activity or total physical activity no conclusion can be drawn regarding the net results in physical activity. Therefore, we cannot tell whether the increase in moderate to vigorous physical activity compensated for the loss of work related activity. Touvier et al. [16] concluded that the increase in overall leisure time physical activity after retirement did not compensate for the loss of work-related activity. Cozijnsen et al. [34] concluded that recent retirees, as compared to older cohorts of retirees, were more likely to report sports participation. More research is needed to systematically assess the effects of retirement on MVPA and sports participation.

The main strength of our study is the use of the longitudinal data. This prospective cohort study allowed us to examine the association of widowhood and retirement with change in physical activity, using the same validated physical activity questionnaire at both time points.

Some limitations should also be mentioned. First, the small number of participants that widowed or retired limited our sample size. A second limitation is the reliance on self-reported data of physical activity. Questionnaires are generally suitable for categorizing or ranking people in levels of physical activity but not accurate enough to assess absolute levels of physical activity [35,36]. Since ranked MVPA scores and dichotomized sports participation we were not able to express the difference between groups in minutes per day. Finally, we have studied the independent influence of two major life events on physical activity. Future research is needed to explore the underlying pathways of how and when major life events influence physical activity.

In conclusion, our findings suggest that the association between major life events and MVPA and sports participation varies by the two major life events under investigation. Widowhood was not associated time spent in MVPA or sports participation, while retirement was associated with higher levels of MVPA, but not sports participation. Research is needed to study the underlying pathways of how major life events are associated with physical activity. Future research needs to assess the effectiveness of physical activity interventions targeting older adults just before or after a life-changing event.

## Competing interests

The authors declare that there are no conflicts of interest.

## Authors’ contributions

All authors make substantial contributions to the design, analysis and interpretation of data. All authors read and approved the final manuscript.
